# Olfactory neuroblastoma: A clinicopathological experience of a rare entity from Pakistan

**DOI:** 10.12669/pjms.323.9738

**Published:** 2016

**Authors:** Saroona Haroon, Muhammad Usman Tariq, Aisha Memon, Saira Fatima, Sheema Habibul Hasan

**Affiliations:** 1 Dr. Saroona Haroon, MBBS, FCPS (Histopathology). Consultant and Histopathology Dept. Chair, Department of Pathology and Laboratory Medicine, Prince Faisal Cancer Centre, King Fahad Specialist Hospital, Buraidah, Kingdom of Saudi Arabia; 2 Dr. Muhammad Usman Tariq, MBBS, FCPS (Histopathology). Instructor, Section of Histopathology, Department of Pathology and Laboratory Medicine, Aga Khan University Hospital, Karachi, Pakistan; 3 Dr. Aisha Memon, MBBS, FCPS (Histopathology). Assistant Professor and Consultant, Section of Histopathology, Department of Pathology and Laboratory Medicine, Aga Khan University Hospital, Karachi, Pakistan; 4 Dr. Saira Fatima, MBBS, FCPS (Histopathology). Assistant Professor and Consultant, Section of Histopathology, Department of Pathology and Laboratory Medicine, Aga Khan University Hospital, Karachi, Pakistan; 5 Dr. Sheema Habibul Hasan, MBBS, FRCPath. Professor and Consultant, Section of Histopathology, Department of Pathology and Laboratory Medicine, Aga Khan University Hospital, Karachi, Pakistan

**Keywords:** Sinonasal tract, Olfactoryneuroblastoma, Esthesioneuroblastoma, Immunohistochemistry

## Abstract

**Objectives:**

To present the clinicopathological experience of Olfactory Neuroblastoma (ONB) with emphasis on histopathological and immunohistochemical features.

**Methods:**

A descriptive cross-sectional study was done on 36 cases of ONB, selected by non-probability purposive sampling. Theses cases of ONB were retrieved and reviewed from surgical pathology database of Aga Khan University Hospital reported between January 1993 and March 2015.

**Results:**

Tumor size and age of presentation was wide in range without any distinct bimodal distribution. Nasal cavity was most common site along with involvement of paranasal sinuses. More than 50% cases had Kadish stage A. Microscopically, most cases were Grade-1 and majority showed partial or complete lobular architecture. Neurofibrillary matrix was observed in 2/3^rd^ of cases. Among immunohistochemical markers, Neuron Specific Enolase was most frequently expressed. Unusual positive expression of Cytokeratin AE1/AE3 and Cytokeratin CAM5.2 was also seen focally in few cases.

**Conclusion:**

The ONB has great variability of histological and clinical presentation, and immunohistochemical markers are useful to differentiate from more common small round blue cell tumours of nasal cavity.

## INTRODUCTION

Olfactory neuroblastoma (ONB), also known as Esthesioneuroblastoma, is a rare but distinct malignant neuroectodermaltumor arising from the olfactory epithelium of roof of nasal cavity and cribriform plate. It account for 2 to 3% of intranasal tumors.^1^

Although rare, but cases primarily arising from other parts of nasal cavity, paranasal sinuses and frontal lobe of brain have also been reported.^2^ These tumors are associated with a number of symptoms including nasal obstruction, epistaxis, headaches, visual disturbances, proptosis, and anosmia.^3^,^4^

Metastasis to distant as well as cervical lymph nodes occurs in 10 to 30% of the cases.^5^ Grossly, they usually appear as a soft, glistening, polypoidal or nodular mass covered by intact mucosa or as friable masses with ulceration and granulation tissue.^6^ Althoughtypical morphologic features of these tumors have been described but most ONBspossess a spectrum of these features which categorize these tumors into 4 grades according to Hyams et al.^7^

In order to correlate the prognosis and progression, different authors have attempted to modify this 4-tiered grading into 2-tiered i.e. high and low grades.^6^-^8^ ONB also have a separate staging system of prognostic value, initially devised by Kadish et al.^9^ and modified by Delguerov et al.^10^ Furthermore, high grade ONB lack classic features and therefore mimic a number of other tumors of sinonasal region with different prognosis and treatment options.^6^

The aim of our study was to observe the spectrum of morphologic features seen in ONB in our region as well as the role of immunhistochemical stains in differentiating them from their mimics.

## METHODS

We retrieved 36 cases of Olfactory neuroblastoma from the surgical pathology database of Section of Histopathology, Aga Khan University Hospital reported between January 1993 and December 2014 through “Integrated Laboratory Management System (ILMS)” software. Since this was a retrospective study and did not involve actual identification of patients, approval from the Hospital Ethics Committee was not required. Clinical information regarding age, sex, location and the presenting complaints was obtained from the pathology reports.

H&E stained microscopic glass slides were reviewed by two pathologists and were analyzed for various histological features including growth pattern, architecture, neurofibrillary matrix, rosettes, nuclear pleomorphism, nucleoli, mitotic activity, necrosis and calcification. Tumors were then graded according to Hyams histologic grade.

The immunohistochemical stains performed were Neuron Specific Enolase (NSE) (Dako,1:400), Chromogranin A (Dako, 1:5000), CD56 (NovoCastra,1:50), Neurofilament (Dako,1:400), S-100 (Dako, 1:1000), Synaptophysin (Biogenex, 1:400), Vimentin (Vim 3B4 Dako, 1:100), CD 99 (mic-2 12E7 Dako, Pre-diluted), Cytokeratin (clone AE1/AE3 Dako 1:50), Epithelial Membrane Antigen (EMA) (E29, Dako, 1:50), Cytokeratin 7 (OV-TL 12/30, Dako, 1:100), CD117 (c-kit – Dako, 1:100), Desmin (D33, Dako, 1:150), Cytokeratin CAM 5.2 (Monoclonal Becton, Dickinson and Company, NJ, Pre-diluted).

Tumors were then staged according to Kadish staging system.^9^ Delguerov stage was not assessed as radiological films were not available in all the cases to accurately evaluate the extent of invasion.

The data was entered in SPSS version 19. The relationship of qualitative clinical and histologic features with tumor grade and grade was analyzed using Chi-square test. For patient’s age and tumor size, the means were compared using one way ANOVA test. *p* value of less than 0.05 was considered positive.

## RESULTS

Out of 36 cases, 20 were excisional biopsies, 12 were incisional biopsies and 4 were received as blocks for 2nd opinion.

Age of presentation ranged from 1-67 years with mean age of 35.58 years +/-18.9 SD. Male to female ratio was 1.4:1. Details of clinical symptoms, site of involvement and stage are given Table-I. No significant association of gender, clinical symptoms and site of involvement was seen with tumor grade or tumor stage. Moreover, no significant increase in tumor stage was observed with higher tumor grade.

**Table-I:** Summary of clinicopathological features of Olfactory neuroblastoma. (n=36).

*Clinicopathological features*	*Expression*
*Gender*	
Male	21 (58.8%)
Female	17 (47.2%)
Symptoms	
Nasal obstruction	17 (47.2%)
Epistaxis	05 (13.8%)
Proptosis	02 (5.6%)
Headache & vomiting	01 (2.8%)
Not specified	16
*Tumor site*	
Nasal cavity	28 (77.8%)
Maxillary sinus	07 (19.4%)
Sphenoid sinus	06 (16.6%)
Ethmoid sinus	05 (13.8%)
Frontal sinus	03 (8.4%)
Infrotemporal fossa	02 (5.6%)
Frontal lobe of brain	01 (2.8%)
*Kadish’s Stage*	
Stage A	21 (58.3%)
Stage B	13 (36.1%)
Stage C	01 (2.8%)

Tumor size ranged from 1.5 cm to 9 cm with average tumor size of 5.25 cm. Interestingly, mean tumor size was significantly higher in Grade-1 tumors (6.5 cms) as compared to mean size of Grade-3 tumors (4 cms) and Grade-4 tumors (2 cms), the *p* value was 0.004. Mean tumor size was not significantly different in tumor grade categories. No statistically significant difference in mean patient’s age at presentation was observed in tumor stage and grade categories.

**Fig. 1:**
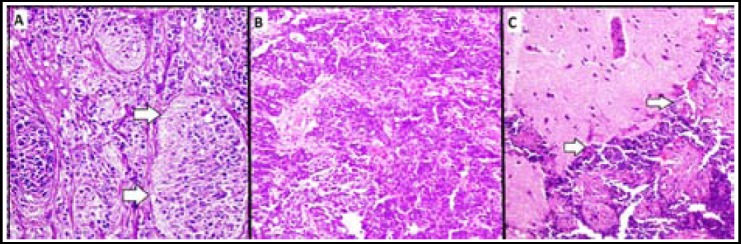
Grade 1 olfactory neuroblastoma arranged in lobules with abundant neurofibrillary matrix and a rim of flattened sustentacular cells at the periphery of lobules. (H&E stain 200x magnification) Grade 4 olfactory neuroblastoma arranged in pattern-less sheets of markedly pleomorphic tumor cells. (H&E stain 200x magnification) Tumor cells invading into the brain tissue. (H&E stain 200x magnification)

Microscopically, the tumors were distributed into all 4 histologic grades with Grade 1 being most common (Table-II). Nuclear pleomorphism was seen in 21 (58.3%) cases and was semi quantitatively assessed to be mild in 7 (19.4%), prominent in 11 (30.6%) and marked in 3 (8.3%) cases. Fig.1(A and B). Similarly, mitotic activity was seen in 25 (70.4%) cases and was semi quantitatively assessed to be mild in 11 (30.6%), prominent in 6 (16.7%) and marked in 8 (22.2%) cases. One of the cases also showed tumour invasion into glial tissue (Fig.1-C). Histological features significantly associated with higher tumor grade included increase in nuclear pleomorphism (*p* value=0.0001), decrease in neurofibrillary matrix (*p* value=0.0001), increased mitotic activity (*p* value=0.0001) and necrosis (*p* value=0.008). Moreover, histological features significantly associated with higher tumor stage included increase in nuclear pleomorphism (*p* value=0.017) and decrease in neurofibrillary matrix (*p* value=0.043).

**Table-II:** Histological features of Olfactoryneuroblastoma. (n=36).

*Hyams histologic grade*	
Grade 1	13 (36.1%)
Grade 2	08 (22.2%)
Grade 3	11 (30.6%)
Grade 4	04 (11.1%)
*Architecture*	
Lobules only	16 (44.4%)
Sheets only	05 (13.9%)
Lobules & sheets	10 (27.8%)
Lobules & trabeculae	01 (2.8%)
Lobules, sheets & Trabeculae	03 (8.3%)
Reticular	01 (2.8%)
Neurofibrillary matrix	24 (66.7%)
Rosettes	14 (38.9%)
Necrosis	07(19.4%)
Calcification	03 (8.3%)
Glands	02 (5.6%)

**Fig.2:**
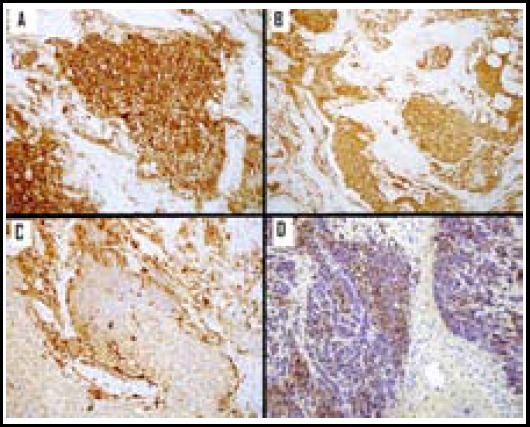
Tumor cells demonstrating diffuse and strong cytoplasmic positivity for Neuron Specific Enolase (NSE) immunohistochemical stain. Tumor cells demonstrating diffuse and strong cytoplasmic positivity for Chromogranin A. S100 immunohistochemical stain highlighting sustentacular cells at the periphery of lobules. Tumor cells demonstrating focal positivity for Cytokeratin AE1/AE3 immunohistochemical stain.

Based on the morphological differential diagnosis of individual case, each case was stained for a variable combination of immunohistochemical stains. Among neuroendocrine markers, Neuron Specific Enolase (NSE) was most frequent expressed, being positive in 18 out of 22 (81.8%) cases. Other immunohistochemical features are tabulated in Table-III and shown in Fig.2.

**Table-III:** Expression of immunohistochemical stains performed.

*ImmunohistochemicalStain*	*No. of cases stained*	*No. of positive cases (%)*
Neuron Specific Enolase	22	18 (81.8%)
Neurofilament	17	10 (58.8%)
Synaptophysin	17	10 (58.8%)
Chromogranin A	21	14 (66.6%)
CD56	13	10 (77%)
Cytokeratin AE1/AE3	24	07 (33.3%)
Cytokeratin CAM 5.2	24	02 (8.3%)
Vimentin	09	05 (55.5%)
S100	16	09 (56.2%)
GFAP	07	02 (28.5%)
CD99 (MIC-2)	11	00
Lymphoid markers	16	00
Desmin	10	00
MyoD1	03	00
HMB45	01	00
TTF-1	01	00
Calretinin	01	00

## DISCUSSION

Histologically, ONB exhibit a spectrum of features on the basis of which they are graded into four categories. ONB typically has a lobular (organoid) pattern with intervening richly vascularized fibrous stroma, which may be hyalinized. Tumor cells are monomorphic with background neurofibrillary matrix. The nuclei contain salt and pepper chromatin. Homer-wright and Flexner-Wintersteiner rosettes are also seen. ONB can demonstrate nuclear atypia, mitosis, calcification and necrosis.^6^,^11^

The entrapment of native minor salivary glands can be misinterpreted as adenocarcinoma. Calcification and gland formation was also seen in few of our cases. (Table-II). Immunohistochemistry is performed to further substantiate the diagnosis and rule out possible mimics, especially in high grade tumors with non-classical features and resembling small round blue cell tumors.

Like morphology, ONB possess a distinct immunohistochemical profile which includes consistently diffuse positivity for neroendocrine/neural markers. The sustentacular cells surrounding the tumors nests demonstrate positivity for S100 immunostain, which might be lost in high grade tumors with sheeting architecture. Approximately one third of the ONB usually demonstrate focal positivity for high as well as low molecular weight keratins (CK AE1/AE3 and CAM 5.2). Similar results are found in our study.^12^-^16^

In 1988, Hyams and co-workers introduced a widely accepted, non-quantitative, 4-tiered grading system based on a constellation of features including growth pattern, presence of neurofibrillary matrix, nuclear atypia, mitotic activity, presence of rosettes and necrosis. This system provides a good overall prediction of outcome but assigning tumor into exact categories can be practically difficult. In order to achieve simplicity and develop correlation of tumor grade with the outcome, several studies have broadly classified ONB into low grade and high grade.^8^,^10^,^17^,^18^

Even Hyams et al.^7^ grouped grade I and II as low grade tumors and grade III and IV as high grade tumors with significant survival difference. In a latest study, Malouf GG et al.^8^ comprehensively studied 30 years data of 44 ONB cases. He evaluated the prognostic significance of various factors and found that high grade ONB (Hyams grade 4) has higher stage at presentation, lower overall survival, lower disease-free survival and higher rates of distant metastasis as compared to low grade ONB (Hyams grade ≤3). Similar results in our study were obtained where recurrence and metastasis was seen in higher grade tumors only.

Common differential diagnosis of ONB include some high grade carcinoma of sinonasal origin including sinonasal undifferentiated carcinoma (SNUC), non-nasopharyngeal carcinoma (NPC), sinonasal neuroendocrine carcinomas (SNEC) and small round blue cell tumors including Ewing’s sarcoma, rhabdomyosarcoma, lymphoma, malignant melanoma.^3^,^14^ Histologically, undifferentiated NPC can mimic ONB if prominent lymphoid background is lacking.^19^

Non-small cell neuroendocrine carcinomas are rare low grade tumors with prognosis better than ONB. Histologically, they resemble ONB is exhibiting an organoid arrangement of tumor cells with low grade morphology. But the intervening stroma is not much fibrovascular as seen in ONB. Immunohistochemically, S100 positive sustentacular cells are not seen as seen in our study in ONB.^20^

Small cell undifferentiated neuroendocrine carcinoma is uncommon tumor of the region which should be differentiated from ONB because of their poor prognosis and different treatment. But they express diffuse positivity for cytokeratins which, if positive, was only focal as per our findings.^21^ Thedistinction from rhabdomyosarcoma relies on the expression of myogenic markers which were negative in all our cases where applied.^6^

Ewing’s sarcoma/Primitive NeuroectodermalTumors (PNET) can pose serious diagnostic challenge in terms of their diverse immunoprofile such as cytokeratin, neuroendocrine marker and occasional S100 positivity in tumor cells. However, CD99 immunostain positivity plays its utmost diagnostic role in these tumors.^15^,^22^

Some ONB can show focal melanin pigment and rare positivity for HMB-45 but positivity for neuroendocrine markers, negativity for Melan-A and careful interpretation of Masson Fontana rules out sinonasal melanoma.^14^,^23^ Lymphoma can show wide spectrum of morphologic features but these neoplasms exhibit a usual diffuse sheet-like growth pattern and positivity for CD45.^6^ Sinonasal Paraganglioma (SP) is also a benign mimic of ONB which also exhibits a nesting pattern of tumor cells with positive neuroendocrine expression and surrounding S100 positive sustentacular cells. The lack of intervening fibrovas cularstroma and tumor location helps to differentiate the two entities.^24^-^26^

Surgical resection of the tumor has ever remained the mainstay of treatment. Initially, the tumors were resected by endoscopic approach but afterwards the craniofacial resection is the operative procedure of choice. Complete surgical resection is more important prognostic factor than tumor stage. Surgical resections seldom yield negative margins. Unfortunately in our study, follow up information was available in 14 cases only, with all the patients having surgical treatment and 11 cases undergoing radiotherapy with 100% five year survival in these patients. Remaining three cases had recurrence, thus substantiating the previous study findings. Therefore, adjuvant or neo-adjuvant radiotherapyhas become the treatment of choice as it provides better control. The role of high-dose chemotherapy has been studied in few studies which have shown an overall longer survival but the benefit is limited in advanced, irresectable and disseminated tumors.^3^

This is the only study with significant number of patients covering detailed histological features, as previously only few case reports of olfactory neuroblastoma have been published from Pakistan. The findings of our study are in concordance with the available literature and effectively summarize them for better understanding the tumor biology. Our study explains the wide morphological spectrum of a rare entity with distinct behavior, outcome and treatment. In depth knowledge of these features will helps distinction from the closely resembling entities and aid in reaching a correct diagnosis. The relationship of morphological features with tumor grade highlights their significance. Moreover, the data from this study points out that small size tumors can have higher tumor and can behave aggressively in future, therefore, warranting close clinical follow up.
